# First Complete Morphological Description of Schistosoma Reflexum in a Llama

**DOI:** 10.1002/vms3.70940

**Published:** 2026-04-03

**Authors:** Carmen Luginbühl, Lea A. Hiller, Elke Van der Vekens, Bettina A. Weber, Cord Drögemüller, Patrik Zanolari, Corinne Gurtner, Joana Jacinto

**Affiliations:** ^1^ Clinic for Ruminants Department of Clinical Veterinary Science Vetsuisse‐Faculty University of Bern Bern Switzerland; ^2^ Institute of Animal Pathology Department of Infectious Diseases and Pathobiology Vetsuisse‐Faculty University of Bern Bern Switzerland; ^3^ Division of Clinical Radiology Department of Clinical Veterinary Science Vetsuisse‐Faculty Bern Switzerland; ^4^ Institute of Genetics Department of Clinical Research and Veterinary Public Health Vetsuisse‐Faculty Bern Switzerland

**Keywords:** congenital malformation, cria, dystocia, New World camelids, South American camelids

## Abstract

Schistosoma reflexum (SR) is a lethal congenital malformation characterized by severe spinal retroflexion, thoracoabdominoschisis with visceral exposure. This case report aims to describe the morphology and pathology of SR in a llama. A white male llama (*Lama glama*) cria was delivered with dystocia following a normal gestation. Pathological examination revealed a severe U‐shaped retroflexion of the spine, thoracolumbar scoliosis and sacral levoscoliosis, abdominoschisis, arthrogryposis, asymmetrical costal malformations, a ventricular septal defect and atresia recti and ani. Computed tomography (CT) of the eviscerated corpus confirmed the severe skeletal dysplasia, including retroflexion and levoscoliosis of the thoracolumbar and sacral spine, a cervical transitional vertebra, costal malformations and multiple angular limb deformities of hindlimbs, as well as bilateral coxofemoral joint subluxation and medial patella luxation on the left side. RT‐qPCR testing excluded teratogenic viral infections. Pathology confirmed the diagnosis of an SR‐type malformation and CT helped to further characterize the skeletal malformations that define SR. This is the first specific morphological description of SR in a llama and highlights the complementary value of CT and postmortem examination for characterizing complex congenital malformations in camelids.

## 1 Background

Congenital malformations, also known as birth defects, are developmental anomalies that originate during gestation and may arise during either the embryonic or foetal stages of development (Mee et al. 2024; Quigley and Mee 2025). In livestock, these abnormalities can involve structural or functional defects that compromise both viability and productivity (Albarella et al. 2017; Quigley and Mee 2025). Some of these disorders are an important cause of dystocia, stillbirth and neonatal mortality, contributing substantially to both animal welfare and economic losses within the livestock industry (Albarella et al. 2017; Agerholm et al. 2025; Quigley and Mee 2025).

The aetiology of congenital malformations is multifactorial, encompassing genetic mutations, chromosomal abnormalities, teratogenic agents, nutritional deficiencies and infectious causes that interfere with normal embryonic and foetal development (Schalles et al. 2014; Agerholm et al. 2015; Jacinto et al. 2024; Mee et al. 2024; Jacinto et al. 2025). Among ruminants like cattle, sheep and goats, common congenital defects include intestinal atresia, schistosoma reflexum (SR), skeletal deformities like ankylosis and neural tube defects such as anencephaly (Bezek and Frazer 1994; Laughton et al. 2005; Marcolongo‐Pereira et al. 2010; Mee et al. 2024).

South American camelids (SACs) exhibit both unique and similar malformations compared to other ruminants. SR in llamas has been estimated to represent 1.4% of reported congenital malformations (Leipold et al. 1994). Frequently described anomalies include choanal atresia, limb and skeletal deformities such as polydactyly and craniofacial or ocular malformations (Tibary et al. 2011).

SR is a lethal syndromic congenital syndrome characterized by a pronounced U‐shaped retroflexion of the spine, accompanied by thoracoschisis and abdominoschisis leading to the externalization of abdominal viscera (Jacinto et al. 2024). Additional anomalies commonly associated with SR include arthrogryposis, ankylosis of the hind limbs, and, less frequently, palatoschisis and other developmental defects (Jacinto et al. 2024). This syndromic malformation is well documented in cattle (OMIA: 000890–9913) and has also been reported in several other domestic species including goats (OMIA: 000890–9925), horses (OMIA: 000890–9796), donkeys (OMIA: 000890–9793), sheep (OMIA: 000890–9940, Tsuma and Abuom 2009), pigs, cats (Mateo and Camón 2008), dogs (Özsoy et al. 2009) and SAC (Leipold et al. 1994). However, to the authors’ knowledge, no detailed morphological description of SR in llamas has been reported. Therefore, the objective of this case report was to describe the morphological and pathological features of SR in a llama.

## 2 Material and Methods

### 2.1 Signalment and History

A white male llama (*Lama glama*) cria was delivered with dystocia following a normal gestation period and died shortly after birth. The dam was a 6‐year‐old grey‐and‐white woolly llama, and the sire a 12‐year‐old white‐and‐black woolly llama. There was no evidence of parental consanguinity within at least four generations. The dam had previously produced two crias, neither of which exhibited congenital malformations; one of these offspring was also sired by the same male. The sire had produced more than 67 crias, with only one additional male offspring (born in the same year as the present case) reported to have thoracolumbar scoliosis and was subsequently euthanized. The dam of that particular offspring had previously delivered seven crias without congenital malformations. The herd had official bovine viral diarrhoea virus (BVDV)‐free status.

### 2.2 Pathological Examination

A complete post‐mortem and morphologic examination were carried out. Tissue samples from the brain, lung, heart, liver and kidney were collected and placed in 4% formalin. The following day, formalin‐fixed tissues were trimmed, embedded in paraffin and haematoxylin and eosin (HE) stained histological tissue sections were prepared. Slides were then assessed under the light microscope.

### 2.3 Computer Tomography

The eviscerated cadaver was placed in U‐shaped positioning cushion with its cervical and cranial thoracic segment in dorsal recumbency. The head and atlas had been removed prior to the scan. A complete computed tomographic examination was performed with a photon counting computed tomographic scanner (Naeotom Alpha, Siemens Healthineers, Zurich, Switzerland) at the Division of Clinical Radiology, Vetsuisse‐Faculty, University of Bern, using 140 kVp and 364 mA, a pitch of 0.9 and a single collimation width of 0.4 mm. The images were reconstructed with a bone and soft tissue kernel in both 0.4 and 1 mm slice thickness. The acquired dataset was stored in DICOM format on the clinical PACS system and reviewed in a dedicated DICOM viewer (DeepUnity Diagnost 2.3.0.0; Dedalus Healthcare GmbH, Bonn, Germany) in using multiplanar reconstructed images and 3D volume renderings.

### 2.4 Teratogenic Virus Testing

To exclude common viral causes of congenital malformations in Switzerland, tissue from the spleen was tested for bluetongue virus (BTV) and Schmallenberg virus (SBV) using reverse transcription quantitative PCR (RT‐qPCR).

## 3 Results

### 3.1 Morphological and Pathological Features

**FIGURE 1:**
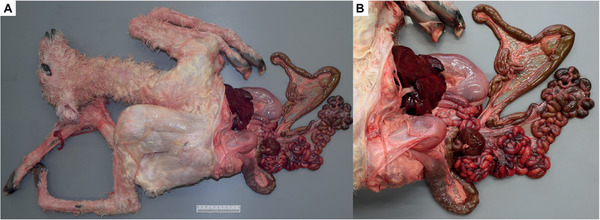
Gross morphology of schistosoma reflexum in a llama cria. (A) General appearance characterized by U‐shaped dorsal to dorsolateral retroflexion of the spine and exposure of the viscera. (B) Particular of the exposure of the viscera.

The cria presented a severely U‐shape retroflexed spine, resulting in near parallel alignment of the sacrum and cervical vertebrae (Figure 1A). Marked left‐sided scoliosis affected the thoracic and lumbar vertebrae. The abdominal wall was incompletely formed (abdominoschisis), leaving the abdominal viscera completely exposed (Figure 1B). All limb joints were moderately to severely contracted and stiffened (arthrogryposis).

The thorax had 12 ribs on both the left and right sides, with an intact diaphragm. On the right side, five ribs had a normal curvature, while the remaining seven ribs were dorsally bent. Due to the severe thoracic malformation and consequent space limitation, the right lung was moderately reduced in size.

Cardiac examination revealed a ventricular septal defect of 1.1 cm in diameter located in the septum directly ventral to the atrioventricular valves. The foramen ovale and the ductus arteriosus were open, as expected in newborn animals. The colon ended blindly and lacked an anus (atresia recti and atresia ani). The penis and intraabdominal testis were present.

Histological examination of the lung revealed multifocal to coalescing ventilated areas. The lumina of bronchioles and bronchi contained abundant keratin squames, with small numbers of neutrophils and fibrinous exudate.

Taken together, the morphological and pathological findings were consistent with an SR‐type congenital malformation.

### 3.2 Computed Tomography‐Based Morphological Findings

**FIGURE 2:**
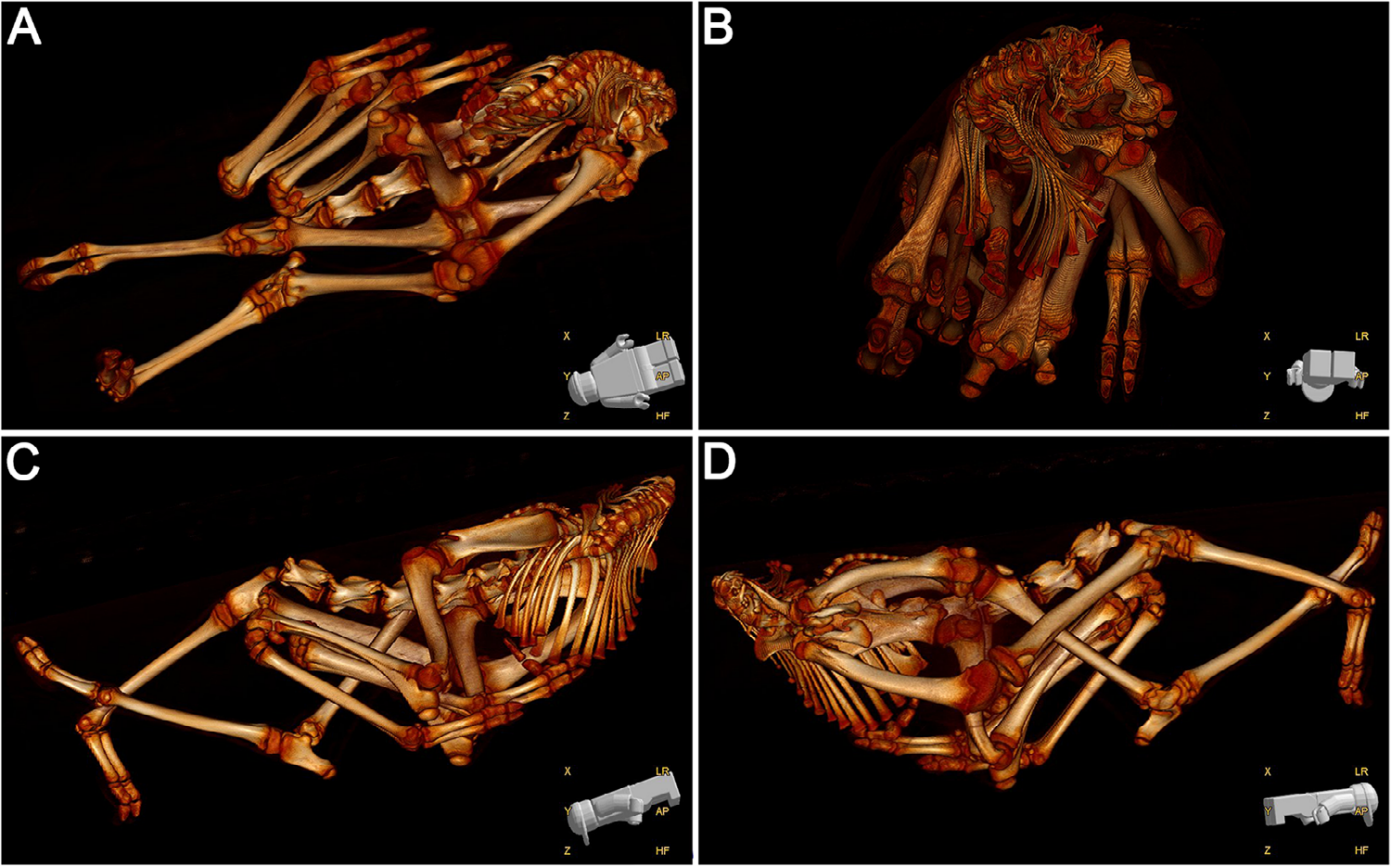
Volume rendered computed tomographic images of a newborn llama cria with schistosoma reflexum. Ventral (A), caudal (B), left (C) and right (D) views are shown to the cervical and cranial thoracic spine, highlighting the U‐shaped dorsolateral retroflexion and levoscoliosis of the spine and the abnormal shape and position of the pelvic limbs. Cranial is to the left (A, C), right (D) or to the back of the figure (B).

The animal was eviscerated and the head and the atlas were removed prior to the CT examination. The six remaining cervical vertebrae were within normal limits, but a short right‐sided rib was present on C7. Twelve pairs of ribs were present, all 12 right and the first 5 left ribs showed a ventral orientation; the left rib on T6 was shortened and showed a dorsal angulation. The left 7th to 12th ribs were dorsally bent. All ribs were to some degree abnormal in shape, but most pronounced caudal to T5 bilaterally and worse on the left. All thoracic and lumbar vertebrae were malformed, many showing a wedge‐shape and lacking endplates. From T4 caudally most vertebral arches were fused and there was a narrowing or near absence of the intervertebral disc spaces. From T6 to T8, there was a rightwards deviation of the spinal column (levoscoliosis) and a mild lordosis, but a marked dorsoflexion as well as levoscoliosis occurred from T11 to L5, showing a 180° U‐turn of the spine (Figure 2). The thoracic spinal malformations primarily affected the vertebral arches rather than the vertebral bodies. This resulted in the long axis of the sacrum being parallel to the cranial thoracic spine but facing opposite directions. L3–L5 were indistinguishable. The sacrum consisted of five fused vertebral bodies, dorsally tapering and showing a dextroscoliosis and lordosis. The sacral wings and arches were markedly distorted, creating an oblique right dorsal‐left ventral rotation of both the iliac and sacral wings.

The pelvis showed a left convex‐right concave pelvic axis curvature with severe pelvic canal narrowing. Bilateral subluxation of the coxofemoral joints was observed with a marked supinated and hyperextended position of both femora. Both femoral heads were misshapen, and the femora showed a valgus and antecurvatum in their diaphysis. The left proximal tibia was endorotated and showed a valgus. A concurrent left‐sided medial patella luxation was present. A mild valgus and exorotation of the right metatarsus and mild varus of the distal aspect of the left metatarsus were observed. The forelimbs exhibited arthrogryposis affecting all joints.

### 3.3 Exclusion of Common Teratogenic Virus

Prevalent teratogenic viruses in Switzerland, which are known to cause congenital malformations in domestic ruminants, were excluded. RT‐qPCR testing of splenic tissue for BTV and SBV were negative for both viruses.

## 4 Discussion

In this case report, we describe the first complete morphological description case of SR in a llama (*L. glama*) cria, characterized by a severe U‐shaped retroflexion, levoscoliosis of the spine with a visceral exposure and arthrogryposis. Additional abnormalities involving the spine, thorax, heart and intestines were identified. Computer tomography provided detailed visualization of the spinal, costal and limb malformations.

SR develops during early embryogenesis, coinciding with critical phases of body wall closure and axial rotation (Windsor 2019). This syndromic condition is morphologically characterized by marked retroflexion of the thoracolumbar spine and the thoracoabdominal schisis associated with abdominoschisis (Jacinto et al. 2024). These severe malformations significantly impair parturition mechanics, predisposing to dystocia—a complication consistently documented in bovine SR cases (Jacinto et al. 2024) and observed in the present llama case.

In addition to abdominoschisis and spinal retroflexion, the main pathological alterations in our case included marked scoliosis of the thoracic and lumbar vertebrae. A ventricular septal defect was also identified, consistent with cardiac malformations previously reported in bovine SR cases (Jacinto et al. 2024). Furthermore, anorectal malformations, specifically atresia recti and ani, were present. These defects have been previously documented in SR‐affected calves (Jacinto et al. 2024) and as isolated congenital malformations in crias (Tibary et al. 2011).

CT imaging proved highly valuable for the in situ evaluation of skeletal and visceral malformations. Similar applications of CT for the characterization of congenital anomalies have been reported in domestic ruminants, emphasizing its diagnostic utility in complex morphologic disorders (Agerholm et al. 2014; Jacinto et al. 2025). The integration of advanced imaging with pathology therefore enhances diagnostic accuracy and facilitates comprehensive documentation of rare congenital conditions in SAC.

Prevalent teratogenic viruses in Switzerland, such as BTV and SBV, were excluded as potential infectious causes of SR based on negative test results. This suggests that the malformation may have resulted from a de novo genetic variant, similar to cases previously reported in cattle (Jacinto et al. 2024). In a future study, whole‐genome sequencing with a trio‐approach could lead to the identification of potential hereditary or de novo variants implicated in the pathogenesis of SR in camelids.

## 5 Conclusion

We report the first complete morphological description SR in a llama (*L. glama*) cria, characterized by severe U‐shaped spinal retroflexion and levoscoliosis, visceral exposure and multiple skeletal and visceral malformations. Computed tomography provided detailed in situ visualization of the skeletal abnormalities, complementing postmortem pathological examination. The absence of prevalent teratogenic viral infections suggests a possible de novo genetic origin, as previously reported in cattle. Future trio‐based genomic analyses may help clarify the molecular basis of SR in camelids. This case highlights the value of integrating imaging, pathology, and genetics to improve understanding of rare, complex congenital malformations.

## Author Contributions

**Carmen Luginbühl**: investigation, writing – original draft preparation, writing – review and editing, visualization. **Lea A. Hiller**: methodology, investigation, writing – review and editing. **Elke Van der Vekens**: methodology, investigation, visualization, writing – review and editing. **Bettina A. Weber**: investigation, writing – review and editing. **Cord Drögemüller**: writing – review and editing, funding. **Patrik Zanolari**: writing – review and editing, funding. **Corinne Gurtner**: conceptualization, methodology, investigation, writing – review and editing, supervision. **Joana Jacinto**: conceptualization, methodology, writing – review and editing, supervision, visualization, project administration. All authors have read and agreed to the published version of the manuscript.

## Funding

Joana Jacinto is supported in part by the Faculty Clinical Research Platform (FCRP) of the Vetsuisse‐Faculty of the University of Bern.

## Ethics Statement

All animals in this study were handled in accordance with the Swiss Animal Welfare Act and institutional ethical guidelines. Collection of blood samples was approved by the Cantonal Committee for Animal Experiments (Canton of Bern; permit BE94/2022).

## Conflicts of Interest

The authors declare no conflicts of interest.

## Data Availability

The authors have nothing to report.

## References

[vms370940-bib-0001] Agerholm, J. S. , M. Hewicker‐Trautwein , K. Peperkamp , and P. A. Windsor . 2015. “Virus‐Induced Congenital Malformations in Cattle.” Acta Veterinaria Scandinavica 57, no. 1: 54. 10.1186/s13028-015-0145-8. 26399846 PMC4581091

[vms370940-bib-0002] Agerholm, J. S. , W. Holm , M. Schmidt , P. Hyttel , M. Fredholm , and F. J. McEvoy . 2014. “ *Perosomus elumbis* in Danish Holstein Cattle.” BMC Veterinary Research 10: 227. 10.1186/s12917-014-0227-2. 25253618 PMC4181705

[vms370940-bib-0020] Agerholm, J. S. , C. Drögemüller , D. J. Steffen , and J. G. P. Jacinto . 2025. “An Overview of Developmental Disorders Leading to Dystocia in Cattle.” Reproduction in domestic animals = Zuchthygiene 60 Suppl 3(Suppl 3): e70083. 10.1111/rda.70083. 40899133 PMC12406085

[vms370940-bib-0003] Albarella, S. , F. Ciotola , E. D'Anza , A. Coletta , L. Zicarelli , and V. Peretti . 2017. “Congenital Malformations in River Buffalo (*Bubalus bubalis*).” Animals 7, no. 2: 9. 10.3390/ani7020009. 28208595 PMC5332930

[vms370940-bib-0004] Bezek, D. M. , and G. S. Frazer . 1994. “Schistosomus Reflexus in Large Animals.” Compendium of Continuing Education for the Veterinary Practitioner 16: 1393–1398.

[vms370940-bib-0006] Jacinto, J. , A. Letko , A. Gentile , et al. 2025. “Exploring Skeletal Disorders in Cattle and Sheep: A WGS‐Based Framework for Diagnosis and Classification.” Genetics, Selection, Evolution 57, no. 1: 51. 10.1186/s12711-025-01002-z. PMC12465177 40999323

[vms370940-bib-0007] Jacinto, J. G. P. , I. M. Häfliger , A. Letko , et al. 2024. “Multiple Independent De Novo Mutations Are Associated With the Development of Schistosoma Reflexum, a Lethal Syndrome in Cattle.” Veterinary Journal 304: 106069. 10.1016/j.tvjl.2024.106069. 38281659

[vms370940-bib-0008] Laughton, K. W. , K. R. S. Fisher , W. G. Halina , and G. D. Partlow . 2005. “Schistosomus Reflexus Syndrome: A Heritable Defect in Ruminants.” Anatomia Histologia Embryologia 34: 312–318. 16159373 10.1111/j.1439-0264.2005.00624.x

[vms370940-bib-0009] Leipold, H. W. , T. Hiraga , and L. W. Johnson . 1994. “Congenital Defects in the Llama.” Veterinary Clinics of North America Food Animal Practice 10, no. 2: 401–420. 10.1016/s0749-0720(15)30572-7. 7953971

[vms370940-bib-0010] Marcolongo‐Pereira, C. , A. L. Schild , M. P. Soares , S. F. Vargas Jr , and F. Riet‐Correia . 2010. “Congenital Defects in Ruminants in Southern Brazil.” Pesquisa Veterinaria Brasileira 30, no. 10: 816–826. 10.1590/S0100-736X2010001000003.

[vms370940-bib-0011] Mateo, I. , and J. Camón . 2008. “Schistosoma Reflexum in a Cat: Insights Into Aetiopathogenesis.” Journal of Feline Medicine and Surgery 10, no. 4: 376–379. 10.1016/J.JFMS.2007.12.010. 18339568 PMC10832896

[vms370940-bib-0012] Mee, J. F. , D. Murphy , and M. Curran . 2024. “Bovine Congenital Defects Recorded by Veterinary Practitioners.” Reproduction in Domestic Animals 59, no. 1: e14501. 10.1111/rda.14501. 37975255

[vms370940-bib-0013] Özsoy, S. Y. , Ç. Oto , and R. Haziroǧlu . 2009. “Schistosoma Reflexum in a Dog.” Ankara Universitesi Veteriner Fakultesi Dergisi 56, no. 3: 225–226. 10.1501/vetfak_0000002220.

[vms370940-bib-0014] Quigley, K. , and J. F. Mee . 2025. “Bovine Congenital Defects Recorded in a National Survey of Dairy and Beef Herds Over Ten Years (2014–2023).” Reproduction in Domestic Animals 60, no. 5: e70067. 10.1111/rda.70067. 40304553 PMC12042788

[vms370940-bib-0015] Schalles, R. R. , H. W. Leipold , and R. L. McCraw . 2014. Congenital Defects in Cattle. Extension Beef Cattle Resource Committee.

[vms370940-bib-0017] Tibary, A. , Y. Picha , and L. K. Pearson . 2011. “Congenital Anomalies in Crias.” https://cabidigitallibrary.org.

[vms370940-bib-0018] Tsuma, V. T. , and T. Abuom . 2009. “A Case Report of Schistosomus Reflexus in a Lamb.” Kenya Veterinarian 32: 45298.

[vms370940-bib-0019] Windsor, P. A. 2019. “Abnormalities of Development and Pregnancy.” In Veterinary Reproduction & Obstetrics, 168–194. Elsevier. 10.1016/B978-0-7020-7233-8.00009-4.

